# Combined Exogenous Activation of Bovine Oocytes: Effects on Maturation-Promoting Factor, Mitogen-Activated Protein Kinases, and Embryonic Competence

**DOI:** 10.3390/ijms242115794

**Published:** 2023-10-31

**Authors:** Cecilia Valencia, Felipe Pérez-García, Luis Aguila, Ricardo Felmer, María Elena Arias

**Affiliations:** 1 Laboratory of Reproduction, Centre of Reproductive Biotechnology (CEBIOR-BIOREN), Faculty of Medicine, Universidad de La Frontera, Temuco 4811322, Chileluis.aguila@ufrontera.cl (L.A.); ricardo.felmer@ufrontera.cl (R.F.); 2 Department of Agricultural Sciences and Natural Resources, Faculty of Agriculture and Forestry Sciences, Universidad de La Frontera, Temuco 4811322, Chile; 3 Department of Animal Production, Faculty of Agriculture and Forestry Sciences, Universidad de La Frontera, Temuco 4811322, Chile

**Keywords:** parthenogenesis, inhibitors, anisomycin, cycloheximide, DMAP, MPF, MAPKs, *CDX2*, *POU5F1*, *BCL2A1/BAX*

## Abstract

Oocyte activation via dual inhibition of protein synthesis and phosphorylation has improved in vitro embryo production in different mammalian species. In this study, we evaluated the effects of the combination of cycloheximide (CHX), dimethyl amino purine (DMAP), and anisomycin (ANY) on the activation of bovine oocytes, particularly on dynamics of MPF and MAPKs, embryonic developmental potential, and quality. The results showed that the cleavage and blastocyst rates, as well as levels of CCNB1, CDK1, p-CDK1^Thr161^, and p-CDK1^Thr14-Tyr15^, were similar among groups; ANY and ANY + CHX reduced the expression of ERK1/2 compared to DMAP-combinations (*p* < 0.05), whereas ANY + DMAP, CHX + DMAP, and ANY + CHX + DMAP reduced p-ERK1/2 compared to ANY and ANY + CHX treatments (*p* < 0.05). The quality of blastocysts in terms of cell counts, their allocation, and the numbers of TUNEL-positive cells did not differ among groups. However, transcript levels of *POU5F1* were higher in embryos derived from ANY + CHX + DMAP treatment compared to other groups, while expression levels of *CDX2* did not show differences. In addition, the *BCL2A1/BAX* ratio of the ANY + CHX + DMAP treatment was significantly low compared to the ANY treatment (*p* < 0.05) and did not differ significantly from the other treatments. In conclusion, oocyte activation by dual inhibition of protein synthesis and phosphorylation induces MPF inactivation without degradation of CCNB1, while MAPK inactivation occurs differentially between these inhibitors. Thus, although the combined use of these inhibitors does not affect early developmental competence in vitro, it positively impacts the expression of transcripts associated with embryonic quality.

## 1. Introduction

Parthenogenesis has been used extensively as a model to study the biochemical and morphological events that occur during the early stages of embryonic development [1]. During the last few decades, one of the most relevant applications has been to boost the developmental potential of embryos generated through intracytoplasmic sperm injection (ICSI) [2] and/or somatic cell nuclear transfer (SCNT) [3]. Additionally, parthenogenesis circumvents ethical and legal problems of research (such as pharmacological or toxicological studies) on human embryos. It offers a valuable tool for characterizing and dissecting the distinctive molecular signatures of the female gamete [4]. Additionally, it represents a potential source of immunocompatible stem cells for the oocyte donors, making it a model highly suitable for stem-cell therapy and/or establishing a histocompatibility biobank [5,6,7]. Based on these factors, parthenotes have been employed in different research areas, ranging from assisted reproduction technologies to basic biology or studying mechanisms underlying embryonic development.

Oocyte activation protocols consist of the use of physical [8] or chemical stimuli [2,9,10,11,12,13,14] to release the female gamete from metaphase II arrest, and subsequent meiotic resumption. The maturation-promoting factor (MPF) complex is the master regulator of both mitotic and meiotic cell cycles. This heterodimer is composed of a regulatory subunit, the cyclin B1 protein (CCNB1), and a catalytic subunit, cyclin-dependent kinase (CDK1) [15,16,17].

During the meiotic cycle of mammalian oocytes, the activity of MPF follows a well-defined pattern. It is low during the prophase of the first meiotic division (MI), and gradually increases as oocytes resume meiosis until reaching its maximum level at MI metaphase. At the transition from MI metaphase to anaphase, MPF is inactivated. Then, as oocytes progress to the metaphase of the second meiotic division (MII), MPF activity increases again and remains high during MII arrest. After oocyte activation, MPF activity decreases sharply, promoting the resumption of the cell cycle [18].

The MPF activity in female gametes cultivated in vitro is affected by various factors, such as the stage of the meiotic cycle, the species, the age and quality of the oocytes, and the oocyte activation protocol used. The molecular mechanisms behind these fluctuations are not yet fully understood.

It has been recently reported that both sperm-induced activation (after IVF) and the chemically induced activation of bovine oocytes inactivate MPF by CDK1-specific phosphorylation without degrading CCNB1 [19]. This is unlike what has been reported in fertilized mouse MII oocytes, where MPF inactivation occurs through the rapid degradation of CCBN1 [20,21].

Therefore, the inhibition of CDK1 seems to be the mechanism by which in vitro matured bovine oocytes can be successfully activated. This is consistent with what was observed by [22,23], who successfully activated bovine oocytes using specific inhibitors of CDK1 kinase (bohemine and sodium pyrophosphate, respectively) in combination with ionomycin.

The activity of MPF is known to depend on the phosphorylation status of CDK1. Previous studies have shown that phosphorylation of both Thr14 and Tyr15 on CDK1 inhibits MPF activity [24,25]. On the other hand, phosphorylation of Thr161 is necessary to keep MPF in an active state [24,25,26]. It is worth noting that CDK1 can also exist in an inactive state, known as pre-MPF, where it is phosphorylated at both inhibitory (Thr14 and Tyr15) and activator (Thr161) residues [27].

The meiotic arrest is also supported by the cytostatic factor (CSF), conformed by the proteins of the mitogen-activated protein kinases (MAPKs) signaling pathway [28]. Evidence suggests that the MAPKs pathway and its downstream substrates directly promote the activation and stabilization of MPF. This is mainly based on research showing that oocytes from knockout mice for the c-MOS gene or with decreased expression by an RNAi are unable to maintain the MII arrest and subsequently activate parthenogenetically [29,30,31]. In the same way, bovine oocytes microinjected with the phosphatase messenger MKP1, which inactivates MAPKs by dephosphorylation, do not show MII arrest, but do show decreased post-maturation MPF activity, as well as disorganized MII spindles with misaligned chromosomes [32]. Likewise, the inhibition of MEK1 by UO126 generates parthenogenic activation and decreased MPF activity in mouse oocytes [33,34] and pig oocytes [35]. In mammalian oocytes, the activity of the MAPKs pathway follows a well-defined pattern that increases after germinal vesicle breakdown (GVBD), reaching its peak in metaphase-II-arrested eggs, and then gradually declines after MII exit [36,37]. These findings suggest that MAPKs play a role in sustaining elevated CDK1 activity during the second metaphase, as CDK1 activity must decrease for MII exit. Therefore, the MAPKs pathway would be essential in mammalian oocytes to maintain the arrest in MII, MPF activity, and spindle organization. Therefore, fluctuations in MPF and MAPKs activity can be used as indicators of the success and efficiency of artificial activation of mammalian oocytes [18].

Previously, we have reported a differential modulation in the activity of MPF between physiologic activation of oocytes through in vitro fertilization (IVF) and chemical activation using protein synthesis inhibitors such as ANY and CHX. Specifically, we determined that the activity of MPF after in vitro fertilization (IVF) is dependent on the activity of ERK1/2 signaling pathways. In contrast, in chemically activated oocytes, the inactivation of MPF occurs independently of ERK1/2 signaling [19].

Current protocols for inducing the resumption of meiosis of bovine oocytes involve a sequential treatment, using calcium ionophore, specifically ionomycin, followed by treatment with protein kinase inhibitors such as 6-dimethylamino purine (6-DMAP), which inhibits protein phosphorylation, or alternatively with protein synthesis inhibitors such as cycloheximide (CHX) [13,38,39,40,41,42], which acts by blocking the translocation reaction of peptidyl transferase in ribosomes, truncating the translation [2]. In this sense, our group has been pioneering the use of anisomycin (ANY), a protein synthesis inhibitor, to activate bovine oocytes. It has boosted the developmental competence of bovine zygotes derived by parthenogenesis, ICSI, and SCNT [43,44], as well as parthenogenetic pig embryos [45]. Moreover, bovine SCNT zygotes activated with ANY exhibited similar quality and fewer aneuploidies than those activated with CHX or dimethyl amino purine (DMAP) [44]. Moreover, the parthenotes activated with ANY or CHX do not activate the JNK and P38 signaling pathways related to cellular damage [19]. With a view to improving the efficiency of the chemical activation of mammalian oocytes, several studies have reported the dual combination of oocyte-activating compounds (cycloheximide and DMAP), particularly in horses [46,47,48], pigs [49,50], buffalos [51], goats [52], and rabbits [53,54]. Thus, the combined use of chemical inhibitors raises a valid strategy for improving oocyte activation efficiency and subsequent embryo development. However, the effect of combinations of oocyte-activating compounds on parthenogenetic development in bovine species has not been evaluated.

The present study aimed to analyze the activation efficiency of bovine oocytes using combinations of the aforementioned inhibitors of protein synthesis and phosphorylation. We examined the developmental potential in vitro, the effects on the dynamics of MPF and the ERK1/2 MAPKs pathway, and the quality of bovine parthenogenetic embryos generated by these combinations.

## 2. Results

### 2.1. The Expression Level of CCNB1 and Profile of CDK1 Phosphorylation

First, we investigated the effects of the different treatments on main cell-cycle regulators MPF and MAPKs.

**Figure 1:**
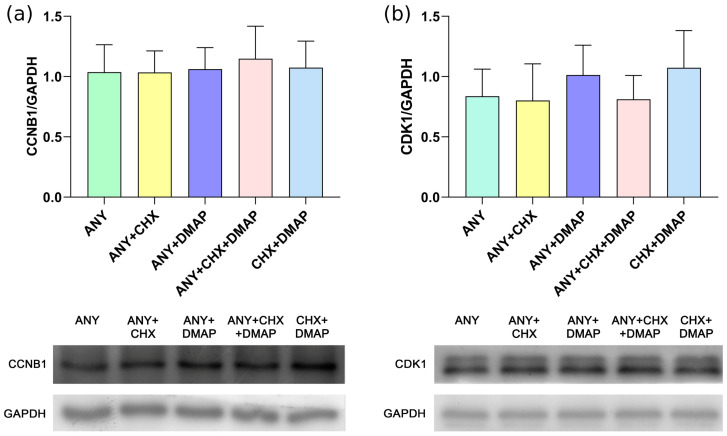
Parthenogenetic activation of bovine oocytes through the combination of protein synthesis and phosphorylation inhibitors does not modify the expression of the MPF components. Groups of 30 oocytes were randomly assigned to different activation treatments consisting of incubation in ionomycin followed by anisomycin (ANY), anisomycin + cycloheximide (ANY + CHX), anisomycin + DMAP (ANY + DMAP), ANY + CHX + DMAP, and CHX + DMAP. All of the treatments included cytochalasin B. The culture was developed for 4 h post-activation. Using a western blot, the following was determined: (**a**) the expression level of total CCNB1 and (**b**) the total level of CDK1. The graphs represent the semi-quantification of the intensity of each band in relation to the load control (GAPDH). The data appear as mean ± standard deviation. ANOVA.

**Figure 2:**
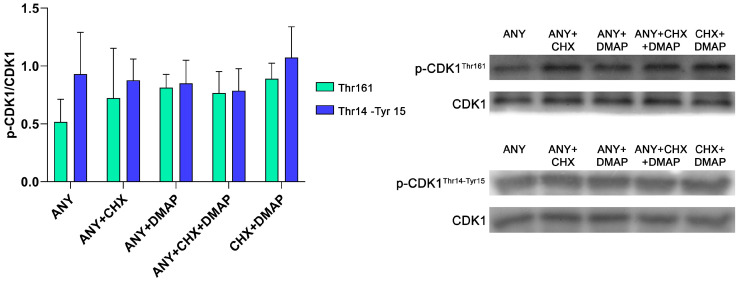
Parthenogenetic activation of bovine oocytes using a combination of protein synthesis and phosphorylation inhibitors inactivates MPF due to high levels of activating and inhibitory phosphorylation (Pre-MPF). Groups of 30 oocytes were randomly assigned to different activation treatments consisting of incubation in ionomycin followed by: anisomycin (ANY), anisomycin + cycloheximide (ANY + CHX), anisomycin + DMAP (ANY + DMAP), ANY + CHX + DMAP, and CHX + DMAP. All of the treatments included cytochalasin B. Cultures were developed for 4 h post-activation. Using western blot, the level of CDK1 kinase-specific phosphorylation was determined in activating residue Thr161 and inhibitory residues Thr14-Tyr15. The graph represents the semi-quantification of the intensity of each band in relation to the load control (CDK1). The data appear as mean ± standard deviation. Two-way ANOVA.

The results indicated that chemical activation methods involving protein synthesis inhibition (ANY and ANY + CHX) or dual inhibition of protein synthesis and phosphorylation (ANY + DMAP, ANY + CHX + DMAP, or CHX + DMAP) did not affect the levels of CCNB1 protein, indicating that the chemical resumption of meiosis II of bovine oocytes does not rely on the degradation or loss of CCNB1. The semi-quantitative analysis is shown in Figure 1a. Similarly, the levels of CDK1 remained constant across all treatments Figure 1b. The data also revealed that there were no differences in the phosphorylation profile of CDK1 for either the activating (p-CDK1^Thr161^) or the inhibitory (p-CDK1^Thr14-Tyr15^) residues among the groups, as represented in Figure 2. Thus, dual inhibition of protein synthesis and phosphorylation (ANY + DMAP, CHX + DMAP, or ANY + CHX + DMAP) generates a phosphorylation profile of CDK1 residues similar to that observed when the activation is carried out only with inhibitors of protein synthesis (ANY or ANY + CHX), and remains phosphorylated on Thr161 and Thr14-Tyr15 residues.

### 2.2. Effect of Parthenogenetic Activation Using Protein Synthesis and Phosphorylation Inhibitors on Main MAP Kinase Proteins

**Figure 3:**
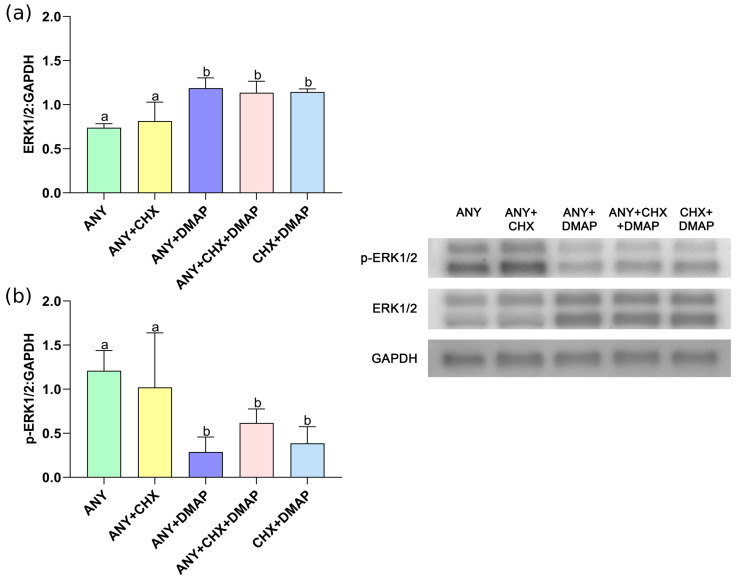
Parthenogenetic activation of bovine oocytes by protein synthesis and phosphorylation inhibitors differentially inactivates the signaling pathway of the kinases ERK1/2. Groups of 30 oocytes were randomly assigned to different activation treatments consisting of incubation in ionomycin followed by: anisomycin (ANY), anisomycin + cycloheximide (ANY + CHX), anisomycin + DMAP (ANY + DMAP), ANY + CHX + DMAP, and CHX + DMAP. All of the treatments included cytochalasin B. Cultures were developed for 4 h post-activation. Using western blot, the following was semi-quantified: (**a**) the level of total expression of ERK1/2 and (**b**) the level of p-ERK1/2. GAPDH was used as the load control in both analyses. The data represent the mean ± standard deviation; n = 3; a/b; *p* < 0.05; ANOVA; the Scheffé post-test.

Expression and profile of ERK1/2 phosphorylation followed. We continued to analyze the total expression of ERK1/2. The data indicated that the use of protein synthesis inhibitors (ANY or ANY + CHX) reduced the expression of ERK1/2 compared to dual inhibition (ANY + DMAP, ANY + CHX + DMAP and CHX + DMAP) (*p* < 0.05); the semi-quantitative analysis is represented in Figure 3a, but it was similar between oocytes activated by protein synthesis inhibitors (ANY and ANY + CHX). In the same way, there were no differences between the groups of oocytes activated by dual inhibition (ANY + DMAP, ANY + CHX + DMAP, and CHX + DMAP), as depicted in Figure 3a.

For the analysis of phosphorylated ERK1/2 (p-ERK1/2), GAPDH was used as a loading control, since protein synthesis inhibitors affect ERK1/2 expression levels. The analysis showed that the dual inhibition decreased the levels of p-ERK1/2 compared to the treatments based only on protein synthesis inhibition (ANY and ANY + CHX) (*p* < 0.05); the semi-quantitative analysis is represented in Figure 3b. The p-ERK1/2 was similar between oocytes activated by ANY and ANY + CHX, as depicted in Figure 3b. In the same way, p-ERK1/2 did not differ among the treatments that combined the inhibition of the protein synthesis and phosphorylation (ANY + DMAP, ANY + CHX + DMAP, and CHX + DMAP), as shown in Figure 3b.

Also, it is important to emphasize that our experimental design, involving membrane division post-protein transfer and incubation with various specific antibodies, allowed us to determine that within the same sample, the activation treatment’s effect can differ among different proteins. This was evident in the treatments containing DMAP (ANY + DMAP, ANY + CHX + DMAP, and CHX + DMAP), which had no effect on CDK1 phosphorylation but had a marked impact on ERK1/2 phosphorylation.

### 2.3. In Vitro Development and Quality of Parthenogenetic Embryos Produced by Combinations of Protein Synthesis and Phosphorylation Inhibitors

Once the effects of the different treatments on main cell-cycle regulators MPF and MAPKs were determined, we evaluated whether the different combinations of inhibitors would impact further embryonic development. The results of 582 oocytes (n = 5) activated using inhibition of protein synthesis or dual inhibition showed no differences in terms of the cleavage (91.9, 91.6, 98.2, 98.6, and 95.6%, respectively) and blastocysts rates (43.2, 37.4, 50.5, 49.6, and 50.9%, respectively), as can be seen in Table 1.

**Figure 4:**
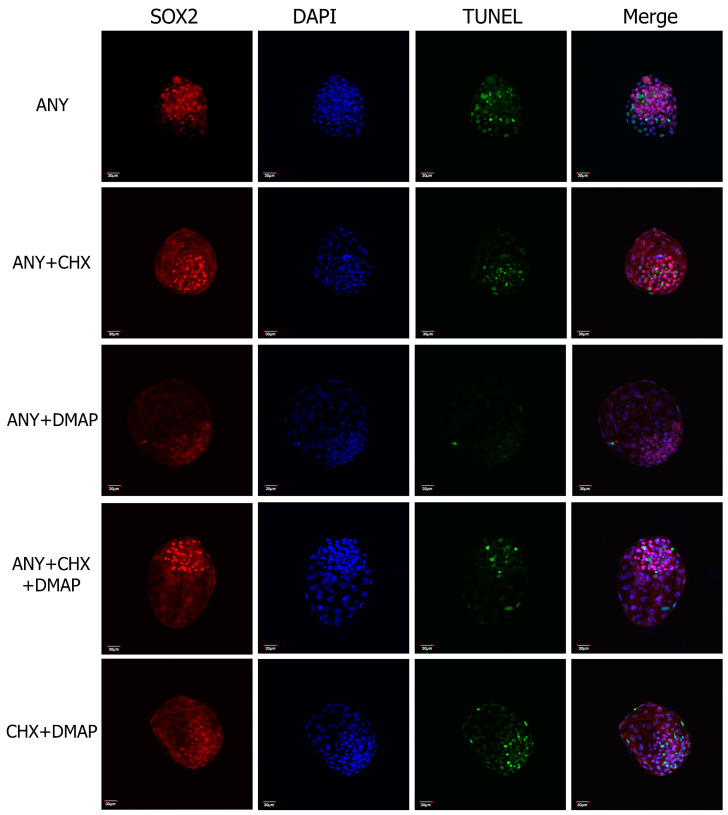
Representative images of differential staining of parthenogenetic embryos produced by combinations of protein synthesis and phosphorylation inhibitors at 168 h of culture. Inner cell mass (ICM) was assessed by immunostaining of SOX2 with a specific marker of ICM (red); number of total cells was assessed by staining with DAPI (blue); TUNEL-positive cells were identified (green); and the merge marked by three qualities of fluorescence, indirectly leading to trophectoderm cells (TE) (purple).

Similarly, the embryo morphology and quality in terms of the total number of cells, allocation to the TE and ICM, and the percentage of cells with fragmented DNA did not show differences among groups; see Table 2, Figure 4.

### 2.4. Effect of the Parthenogenetic Activation on the Expression of Genes Associated with Embryonic Quality

**Figure 5:**
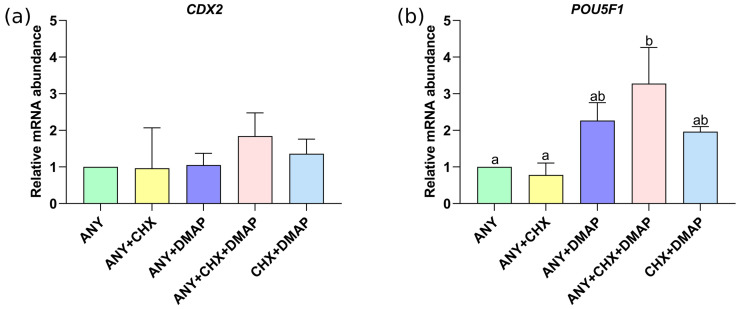
Effect of the combined use of the protein synthesis and phosphorylation inhibitors on the expression of genes related to embryonic development, quality, and differentiation. Using qRT-PCR, the relative abundance of mRNA of *CDX2* (**a**) and *POU5F1* (**b**) was measured in expanded blastocysts obtained by different activation treatments, namely, ionomycin followed by: anisomycin (ANY), anisomycin + cycloheximide (ANY + CHX), anisomycin + DMAP (ANY + DMAP), ANY + CHX + DMAP, or CHX + DMAP. The data represent the mean ± standard deviation, n = 3 biological replicates corresponding to a pool of 5 expanded blastocysts/treatment (15 total embryos per treatment); a/b, *p* < 0.05 compared to the ANY calibrator group. ANOVA, the Scheffé post-test.

The relative abundance of the *CDX2* transcript was similar among treatments; see Figure 5a. By contrast, the levels of the pluripotency-related gene *POU5F1* were higher under activation by ANY + CHX + DMAP, compared to the activation with protein synthesis inhibitors (ANY and ANY + CHX) (*p* < 0.05); see Figure 5b. However, it was similar between oocytes activated by protein synthesis inhibitors (ANY and ANY + CHX); see Figure 5b. In the same way, there were no differences between oocytes activated by dual inhibition (ANY + DMAP, CHX + DMAP, and ANY + CHX + DMAP); see Figure 5b.

**Figure 6:**
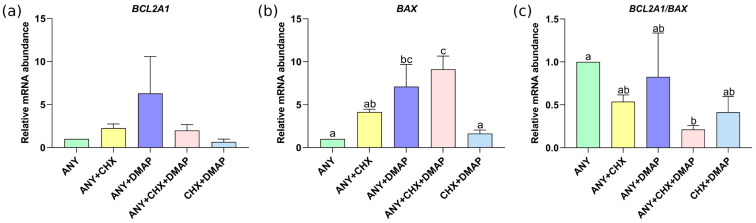
Effect of the combined use of protein synthesis and phosphorylation inhibitors on the expression of genes related to apoptosis. Using qRT-PCR, the relative abundance of mRNA of *BCL2A1* (**a**) and of *BAX* (**b**), as well as the *BCL2A1/BAX* ratio (**c**) were measured in expanded blastocysts obtained by different activation treatments, namely, ionomycin followed by: anisomycin (ANY), anisomycin + cycloheximide (ANY + CHX), anisomycin + DMAP (ANY + DMAP), ANY + CHX + DMAP or CHX + DMAP. The data represent the mean ± standard deviation, n = 3 biological replicates corresponding to a pool of 5 expanded blastocysts/treatment (15 total embryos per treatment); different letters are significantly different, *p* < 0.05 compared to the ANY calibrator group. ANOVA, Scheffé post-test.

On the other hand, the levels of the anti-apoptotic gene *BCL2A1* were high in embryos obtained by inhibition using ANY + DMAP compared to those of the other treatments, but it was not statistically significant; see Figure 6a. The relative abundance of the pro-apoptotic gene *BAX* showed lower levels in embryos obtained under activation with ANY and dual inhibition with CHX + DMAP compared to the embryos obtained under activation with ANY + DMAP and ANY + CHX + DMAP treatments (*p* < 0.05), but the levels were similar to those of the ANY + CHX treatment. The treatment ANY + CHX + DMAP showed the highest level of *BAX* transcript, which was only similar to those of the ANY + DMAP treatment; see Figure 6b. However, the ratio of *BCL2A1* to *BAX* (*BCL2A1/BAX*) showed a significative low level in embryos activated by ANY + CHX + DMAP only when compared to the ANY group (*p* < 0.05); see Figure 6c. The treatments ANY + CHX, ANY + DMAP, and CHX + DMAP showed a level intermedium of *BCL2A1/BAX* ratio and were not significatively different from the ANY or ANY + CHX + DMAP treatments.

## 3. Discussion

In the current study, we utilized combinations of protein synthesis and phosphorylation inhibitors to induce parthenogenetic activation of bovine oocytes. Our data demonstrated that neither inhibition of protein synthesis nor dual inhibition of synthesis and phosphorylation of proteins affected the developmental potential of the oocytes in vitro, up to the blastocyst stage. We focused specifically on the proportions of cleaved embryos and total blastocysts, which are critical indicators of successful embryonic development. Cleaved embryos are those that have undergone the first cell division, while total blastocysts include early, expanded, and hatched stages of development. By comparing the proportions of these different stages of development, we aimed to gain insight into the overall quality of the embryonic development process. However, our analysis revealed no significant differences in the proportions of cleaved embryos and total blastocysts, indicating that there were no variations in embryonic development between the groups being compared. Previous studies in other species have reported that combined inhibition of protein synthesis and phosphorylation improved in vitro developmental potential [50,51,52,53,54,55]. They were even able to produce offspring in horses using somatic cell nuclear transfer [47,48]. It is noteworthy that the embryo developmental rates obtained in our study were consistent with the literature on bovine IVF for both cleavage (range 80–90%) and blastocyst rates (range 25–40%) [3,56,57]. Therefore, our results demonstrate that parthenogenetic activation of bovine oocytes by inhibition of protein synthesis or dual inhibition of synthesis and phosphorylation of proteins generates high cleavage and blastocyst formation rates, comparable to those results achieved by conventional IVF. Overall, our study suggests that these strategies can effectively enhance the in vitro developmental potential of bovine oocytes. Further analysis of the inactivation pattern of cell-cycle regulators MPF and MAPKs showed that chemical activation of bovine oocytes by inhibition of protein synthesis or dual inhibition is not associated with the decrease or loss of CCNB1 protein. This result corroborates a previous report which showed that the inactivation of MPF induced either by the sperm after IVF or by chemical induction does not rely on the degradation or reduction of the CCNB1 protein [19]. Moreover, these findings agreed with the study reported by [58], indicating that parthenogenetic activation of *Xenopus* oocytes induced by phosphorylation inhibition (using DMAP) was characterized by the constant presence of CCNB1 protein. Likewise, the expression of the catalytic subunit of MPF, CDK1 kinase, is not affected by any of the inhibitors tested in this study. Conversely, the analysis of the phosphorylation on the activating (CDK1^Thr161^) and inhibitory (CDK1^Thr14-Tyr15^) residues demonstrated that the patterns of phosphorylation induced by all treatments evaluated in this study were consistent with the findings of our previously reported study [19]. Thus, the phosphorylation pattern observed on CDK1 suggests that oocyte activation is linked to an inactive state of MPF, known as pre-MPF, due to the presence of phosphorylation on both activating residue (Thr161) and inhibitor residues (Thr14-Tyr15) [27]. It is noteworthy that the combined use of protein synthesis inhibitors (ANY plus CHX) did not exhibit any synergistic effects, possibly because both inhibitors interfere with protein translation at the same level.

Furthermore, it is worth noting that the phosphorylation levels at residues pCDK1^Thr161^ or pCDK1^Thr14-Tyr15^, generated by the ANY treatment at 4 h post-activation, align with the phosphorylation pattern previously reported by [19] at the same time of analysis. However, when an inhibitor of phosphorylation (DMAP) was added in combination with a protein synthesis inhibitor (ANY and/or CHX), we observed that CDK1 remained phosphorylated at residues CDK1^Thr161^ or CDK1^Thr14-Tyr15^. In this context, owing to the broad-spectrum nature of DMAP as a kinase inhibitor, it is likely that it also has the potential to deactivate specific kinases with intricate inactivation mechanisms, such as CDKs. For instance, Ref. [59] reported that DMAP reduced MPF activity in in vitro matured Xenopus oocytes, associated with an increase in the phosphorylation level of tyrosine residues on p34cdc2 (now known as CDK1). However, their experimental design focused solely on tyrosine phosphorylation, whereas our study provides a more detailed examination. We employed a specific antibody targeting CDK inhibitory residues, including Tyrosine 15. Importantly, our study reports for the first time the combined effect of major bovine oocyte-activating compounds on CDK1 phosphorylation, encompassing both activating and inhibitory residue phosphorylation.

The dynamics of the inactivation of major MAPKs revealed that the inhibition of the protein synthesis decreased ERK1/2 expression, consistent with the previous literature [19]. Interestingly, the reduction in ERK1/2 following ANY and ANY + CHX treatments was perturbed by the addition of the protein kinase inhibitor DMAP. This suggests that DMAP may have other targets involved in early development. Nevertheless, the limited available information on the proteome of early bovine embryos restricts our ability to associate potential regulators of ERK1/2. Hence, further research is necessary to fully comprehend these findings. Additionally, our results demonstrate that the addition of DMAP to protein synthesis inhibitor treatments reduces p-ERK1/2 levels, consistent with the reports of [59]. It is worth highlighting that the presence of one or both protein synthesis inhibitors does not interfere with the effect of DMAP on p-ERK, which is a significant finding. Therefore, the treatments containing DMAP (ANY + DMAP, ANY + CHX + DMAP, and CHX + DMAP) had no effect on CDK1 phosphorylation, but had a marked impact on ERK1/2 phosphorylation.

Finally, the analysis of embryo quality carried out on the blastocyst produced parthenogenetically showed, in terms of the total number of cells, their cell allocation (MCI or TE), and the presence of DNA damage (number cell TUNEL+). The single or dual inhibition to activate the bovine oocyte did not impact the quality of the parthenogenetic embryos obtained in vitro. These results can be largely explained by the theoretical condition of diploidy of the parthenotes analyzed here, given the use of cytochalasin B in all the parthenogenetic activation treatments, which differs from what was observed by [60], who reported a significant reduction in the number of cells total per embryo and the number of cells contained in the trophectoderm (TE) from bovine-expanded blastocysts derived from oocytes activated with 6-DMAP-ionomycin (haploid embryos), compared to zygotes obtained by IVF (diploid embryos).

In addition, we evaluated the relative abundance of the transcription factor caudal-related homeobox 2 *CDX2*, a known marker of trophectoderm (TE) differentiation [61,62,63]. Importantly, bovine blastocysts with increased *CDX2* expression have been associated with enhanced embryo developmental competence and quality [64,65], and improved pregnancy rates after embryo transfer [66]. Similarly, the pluripotency marker *POU5F1* (also known as *OCT4*) is also associated with embryo quality and developmental competence [67,68,69]. The analysis of *CDX2* expression indicated similarities among groups. However, when oocytes were activated through the inhibition of both protein synthesis and phosphorylation using ANY + CHX + DMAP, the transcript levels of *POU5F1* increased. This could indicate a higher level of embryonic competence. It is worth noting that [60] demonstrated that the activation of bovine oocytes through DMAP-Ionomycin (inhibition of protein phosphorylation) led to a downregulation of *POU5F1* gene expression with respect to IVF zygotes. These results indicate that progression to the blastocyst stage of diploid parthenogenetic bovine embryos is not affected by the oocyte activation protocol, regardless of the selected chemical cocktail.

On the other hand, a higher expression of *BCL2A1* has been documented in oocytes and embryos of good quality, unlike the *BAX* transcript. [70,71]. Additionally, the literature indicates that the evaluation of the expression level of these genes by themselves is not entirely informative; it has been suggested that the *BCL2A1/BAX* ratio is a better marker of cell survival or death [71,72]. Therefore, the low *BCL2A1/BAX* ratio observed for the ANY + CHX + DMAP would suggest a possible pro-apoptotic state in parthenotes derived from oocytes activated with the combination of the three inhibitors. However, this condition was inconsistent with the enhanced apoptotic phenotype in the parthenogenetic embryos. These findings call for further investigation.

## 4. Materials and Methods

Unless otherwise indicated, all of the chemical agents were acquired from Sigma (St Louis, MO, USA).

### 4.1. Collection of Ovaries, Selection, and Maturation In Vitro

The ovaries were collected in the local slaughterhouse (Frigorífico Temuco, Temuco, Chile). The cumulus–oocyte complexes (COCs) were aspirated from follicles between 2 and 7 mm. Groups of 50 oocytes were matured in vitro for 24 h in maturation medium TCM-199 containing 6 μg/mL LH, 6 μg/mL FSH, and 1 μg/mL estradiol, supplemented with 10% (*v*/*v*) bovine fetal serum (HyClone Laboratories, Inc., Logan, UT, USA), and incubated at 38.5 °C in 5% (*v*/*v*) CO_2_ and saturation humidity.

### 4.2. Oocyte Activation and Embryo Culture

After 24 h of maturation, the oocytes were denuded by vortexing in 1 mg/mL hyaluronidase and selected by the presence of the first polar body. The metaphase II (MII) oocytes were assigned randomly to the different activation treatments, consisting of incubation with 5 µM of ionomycin (Calbiochem, San Diego, CA, USA) for 5 min followed by incubation in potassium simplex optimization medium (KSOM; EmbryoMax^®^, Millipore Corp, Billerica, MA, USA) supplemented with (i) 1 μg/mL anisomycin (ANY treatment), (ii) 1 μg/mL anisomycin, plus 10 μg/mL cycloheximide (ANY + CHX treatment), (iii) 1 μg/mL anisomycin, plus 1.9 mM 6-dimethylaminopurine (ANY + DMAP treatment), (iv) 1 μg/mL of anisomycin, plus 10 μg/mL cycloheximide, plus 1.9 mM of 6-dimethylaminopurine (ANY + CHX + DMAP treatment) or (v) 10 μg/mL cycloheximide, plus 1.9 mM 6-dimethylaminopurine (CHX + DMAP treatment) for 5 h. All groups included cytochalasin B (5 µg/mL), a blocker of the formation of contractile microfilaments, to avoid extrusion of the second polar body and, by this, to derivate diploid zygotes which behave similarly to IVF embryos in terms of developmental potential [73,74]. After activation, the oocytes were cultured in 50 μL drops of KSOM medium supplemented with 1% (*v*/*v*) BME essential amino acids, 1% (*v*/*v*) MEM non-essential amino acids, and 4 mg/mL of fatty acid-free bovine serum albumin (BSA). The culture was carried out at 38.5 °C in a gas mixture of 5% CO_2_, 5% O_2_, 90% N_2,_ and saturation humidity [75].

The cleavage rate was recorded on day three of culture or 72 h post activation (hpa), the point at which the embryos were supplemented with 5% FBS. The embryos were cultured until day seven to record the rate of blastocyst formation.

### 4.3. Western Blotting

Analysis of western blotting was performed as previously reported [19]. Briefly, at 4 h after the activation treatment, groups of 30 oocytes were assigned per treatment and washed five times in DPBS 1× (HyClone Laboratories, Inc., Logan, UT, USA) supplemented with 5% (*m*/*v*) polyvinyl alcohol (PVA). Then, the oocytes were lysed by boiling for 5 min in sample buffer SDS 2× containing 5% 2-mercaptoethanol and stored at −80 °C until electrophoresis. Electrophoresis was performed in polyacrylamide gel and 12% sodium dodecyl sulfate (*m*/*v*) (SDS-PAGE), and then the proteins were transferred to polyvinylidene difluoride (PVDF) membranes (Millipore, Bedford, MA, USA). All the antibodies used were diluted in a buffer blot composed of TBS 1×, 0.1% (*v*/*v*) Tween-20, and 1% (*m*/*v*) BSA free of proteases, fatty acids, and globulins.

The membranes were divided into two parts and subsequently incubated with the respective antibodies according to the molecular weight of the target proteins, with the primary antibodies for CCNB1 (55 Kda) (Thermo Fisher Scientific, Rockford, IL, USA, MA5-14319, 1:500), p-CDK1 (34 kDa) phospho Thr161 (Abcam, Boston, MA, USA ab208915, 1:500), p-CDK1 (34 kDa) phospho Thr14, Tyr15 (Thermo Fisher Scientific, Rockford, IL, USA, 701808, 1:500), or p-ERK1/2 (42/44kDa) (CST, Danvers, MA, USA, 4370, 1:1000), followed by incubation with their respective conjugated secondary antibody with horseradish peroxidase (HRP) (1:2000) (CST, Danvers, MA, USA, 7074 and 7076). In the same membranes after stripping and re-testing with the respective antibodies were detected; CDK1 (34 kDa) (Thermo Fisher Scientific, Rockford, IL, USA, MA1-19057, 1:1000), ERK1/2 (42/44 kDa) (CST, Danvers, MA, USA, 4695, 1:1000) and GAPDH (37 kDa) (Thermo Fisher Scientific, Rockford, IL, USA, 437000, 1:2000). 

The immunoreaction was detected using the Westar Supernova chemiluminescence kit (Cyanagen, Bologna, Italy) according to the manufacturer’s instructions, and viewed using autoradiography development (Amersham, Buckinghamshire, UK). The semi-quantification of the relative intensity of the CCNB1:GAPDH, CDK1:GAPDH, p-CDK1:total CDK1, p-ERK1/2:GAPDH, and ERK1/2:GAPDH ratios were quantified by means of optical densitometry of the bands using a free plug-in available for ImageJ 1.53t software (National Institutes of Health, Bethesda, MD, USA) 

The experiment was performed in 3 different biological replicates corresponding to independent ovary collections on different days.

### 4.4. Total Number of Cells, Cell Allocation, and TUNEL Staining

Differential staining was used to assess the total number of cells with DAPI staining and their allocation to the inner cell mass cells (ICM) by immunodetection of SOX2, and indirectly to trophectoderm (TE), by counting SOX2-non-expressing cells in expanded blastocysts generated by parthenogenesis (3 biological replicates, 8 total embryos per treatment), using a free plug-in available for ImageJ 1.53t software (National Institutes of Health, USA) 

Immunostaining was performed as has been described previously [76], with a few modifications. Briefly, the embryos were collected and washed in PBS/PVA and fixed with 4% paraformaldehyde for 15 min and permeabilized with D-PBS with 0.1% sodium citrate containing 1% Triton X-100 for 30 min. After blocking for 2 h in D-PBS with 0.1% Triton X-100, 1% BSA, and 5% goat serum (Gibco, Auckland, New Zealand), embryos were placed in a primary antibody solution, consisting of blocking buffer, a mouse antibody anti-SOX2 (Abcam ab10005 at 1:500), or isotype control antibodies, overnight at 4 °C. After washing 3 times for 10 min and 3 times for 20 min each, embryos were incubated with secondary antibody (1:2000) Alexa Fluor 633-conjugated goat anti-mouse IgG (Life Technologies, Eugene, OR, USA, cat. # A-21052) at RT for 1 h. Embryos were then washed 3 times for 10 min and 3 times for 20 min each. For the TUNEL assay, embryos were incubated with labeling reagent according to the manufacturer’s instructions (In Situ Cell Death Detection Kit, Fluorescein, Roche Applied Science, Indianapolis, IN, USA). A positive control for TUNEL was carried out by treating embryos with 75.4 U DNase I for 15 min at 37 °C before the TUNEL assay, and a negative control was attained by incubating embryos with the fluorescent labeling reagent in the absence of the terminal transferase dUTP enzyme. Finally, embryos were mounted onto a glass slide with Prolong Gold Antifade with DAPI (Life Technologies, Eugene, OR, USA, cat. # P36935) and evaluated using confocal microscopy (Olympus FV 1000 laser-scanning confocal microscope). This experiment was performed in 3 different biological replicates corresponding to independent ovary collections on different days.

### 4.5. RNA Extraction, Reverse Transcription, and Gene Expression Analysis

To analyze gene expression, groups of five Day-7 blastocysts from each treatment were lysed in 20 mL of extraction buffer (XB; Arcturus, Carlsbad, CA, USA) by incubation at 42 °C for 30 min, followed by centrifugation at 3000× *g* for 2 min, and then stored at −80 °C until used. Total RNA was extracted from each pool of embryos (n = 3 biological replicates corresponding to a pool of 5 expanded blastocysts/treatment; 15 total embryos per treatment) using the PicoPure RNA Isolation Kit (Arcturus, Carlsbad, CA, USA) according to the manufacturer’s instructions. Residual genomic DNA was removed by DNase I digestion using the RNase-Free DNase Set (Qiagen, Valencia, CA, USA). Reverse transcription was carried out using the RevertAid H Minus First Strand Kit (Thermo Scientific Inc., Pittsburgh, PA, USA).

To quantify the expression of six developmentally important genes (Table S1), real-time quantitative PCR (qRT-PCR) was performed using the Brilliant II SYBR Green QPCR Master Mix (Stratagene Agilent Technologies, Inc., Santa Clara, CA, USA) in an MX3000P thermocycler (Stratagene Agilent Technologies). After each PCR run, dissociation curves were performed to ensure the amplification of a single PCR product. Negative controls consisting of the same mix, but substituting the cDNA with water were run in parallel with the samples.

The ANY blastocysts were used as calibrators and the values are presented as n times relative difference with respect to the calibrator. The MXPro-MX300P Version 4.10 program (Stratagene Agilent Technologies) was used to quantify the relative mRNA expression levels based on the ΔΔCt method and using the amplification efficiency of each gene as a correction factor [70,77]. The geometric average of HMBS and SF3A1 was used as a reference after analysis with the geNorm Visual Basic program version 2108 (Microsoft Excel).

### 4.6. Statistical Analysis

We performed descriptive statistics based on mean and standard deviation, calculated for each of the analyzed variables using Stat Graphics Plus 5.1 software (StatPoint Technologies Inc., Warrenton, VA, USA, EE. UU). Each experiment was repeated at least three times with different biological replicates, in which each biological replicate corresponded to independent ovary collections on different days.

Raw data were transformed to ratio from the baseline condition.

For calculations of relative quantification of CCNB1, CDK1, ERK1/2, p-ERK1/2, cell allocation analysis, the abundance of mRNA of *CDX2, POU5F1, BCL2A1, BAX, BCL2A1/BAX* ratio, and TUNEL assay, differences between treatments in each experiment were determined by one-way ANOVA. In cases where significant differences were observed, Schffe’s test was used to assess the magnitude of the differences.

In order to determine whether there were significant differences in the proportions of cleaved embryos and total blastocysts, we utilized a chi-squared test with Bonferroni’s correction. Our analysis was conducted using the Statgraphics Centurion XV software, version 15.1.02.

The analysis of variation of the levels of phosphorylation of activating and inhibitory CDK1 residue was performed using GraphPad Prism 7.0 software (GraphPad Software, La Jolla, CA, USA), using two-way ANOVA prior to confirmation of the statistical assumptions of normality and homoscedasticity of variance, followed by Tukey’s multiple comparison test.

## 5. Conclusions

In conclusion, the inactivation of MPF in oocytes activated parthenogenetically by dual inhibition of protein synthesis and phosphorylation is associated with phosphorylation in both activating and inhibitory CDK1 residues (pre-MPF) and constant levels of CCNB1. Moreover, the inactivation of MAPKs differs between inhibitors; the inhibition of protein synthesis reduces the expression of ERK1/2, whereas the dual inhibition inactivates MAPKs by dephosphorylation of ERK1/2. Importantly, the combined inhibition of protein synthesis and phosphorylation does not affect further in vitro development of parthenogenetic bovine embryos. In order to obtain a better understanding, future studies will be aimed at a more in-depth analysis involving global transcriptomic analysis in activated oocytes.

To summarize, our findings indicate that utilizing these inhibitor combinations may enhance the effectiveness of reproductive techniques like intracytoplasmic sperm injection (ICSI) or somatic cell nuclear transfer (SCNT).

## Figures and Tables

**Table 1:** Cleavage and development of bovine parthenotes subjected to different activation treatments. Cleavage was recorded at 72 h of culture and blastocyst rates were recorded at 168 h. ^†^ Percentages are based on the total number of blastocysts.

				Blastocysts							
Treatments	Oocytes (n)	Cleavage (%)		n	Percentage per Cultured Embryo	Early Blastocysts (%) ^†^		Expanded Blastocysts (%) ^†^		Hatched Blastocysts (%) ^†^	
ANY	111	102	(91.9)	48	(43.2 ± 3.0)	22	(45.8)	21	(43.8)	5	(10.4)
ANY + CHX	107	98	(91.6)	40	(37.4 ± 3.3)	16	(40.0)	22	(55.0)	2	(5.0)
ANY + DMAP	111	109	(98.2)	56	(50.5 ± 11.0)	25	(44.6)	25	(44.6)	6	(10.7)
ANY + CHX + DMAP	139	137	(98.6)	69	(49.6 ± 14.5)	35	(50.7)	30	(43.5)	4	(5.8)
CHX + DMAP	114	109	(95.6)	58	(50.9 ± 14.7)	30	(51.7)	26	(44.8)	2	(3.4)

**Table 2:** Effect of the different activation treatments on the total number of cells (Total), inner cell mass (ICM) cells, trophectoderm (TE) cells and TUNEL-positive cells.

Treatments	Mean Cell Number (±SD)				TUNEL Staining
	Total	TE	ICM	ICM: Total (%)	TUNEL-Positive Cells: Total Cells (%)
ANY	100.9 ± 43.0	70.7 ± 25.3	27.9 ± 14.3	26.7 ± 9.7	3.9 ± 2.8
ANY + CHX	118.0 ± 28.9	71.0 ± 21.1	38.3 ± 13.5	34.4 ± 8.7	3.6 ± 1.9
ANY + DMAP	118.1 ± 34.2	96.0 ± 28.0	49.0 ± 17.0	33.6 ± 9.5	2.4 ± 1.9
ANY + CHX + DMAP	130.0 ± 38.7	92.8 ± 22.3	31.2 ± 16.4	24.3 ± 8.3	3.5 ± 1.9
CHX + DMAP	129.8 ± 28.5	108.6 ± 20.7	39.9 ± 7.5	27.2 ± 5.6	4.2 ± 2.1

## Data Availability

The data supporting the findings of this study are available from the corresponding author upon reasonable request.

## Supplementary Materials

The following supporting information can be downloaded at: https://www.mdpi.com/article/10.3390/ijms242115794/s1.
