# Supplementary material for: Combined Exogenous Activation of Bovine Oocytes: Effects on Maturation-Promoting Factor, Mitogen-Activated Protein Kinases, and Embryonic Competence
Source: Int J Mol Sci. 2023 Oct 31;24(21):15794. doi: 10.3390/ijms242115794 (PMC10649646; doi:10.3390/ijms242115794)
Supplement: Supplementary file 1 [file ijms-24-15794-s001.zip › ijms-2615435-Supplementary Table S1.pdf]

CDX2

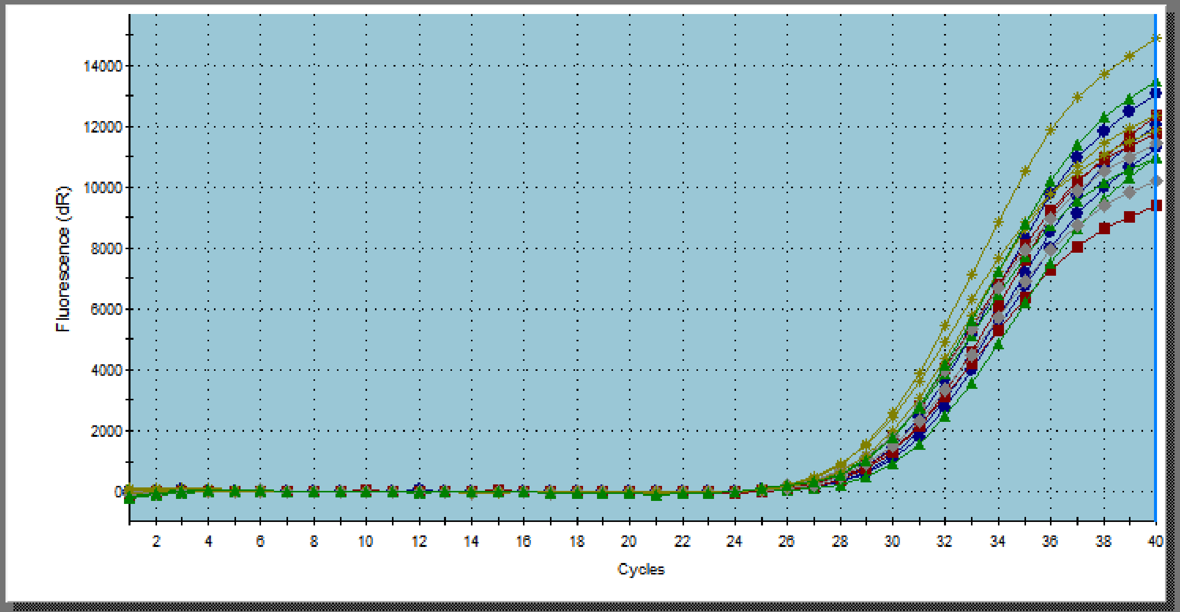

OCT.4

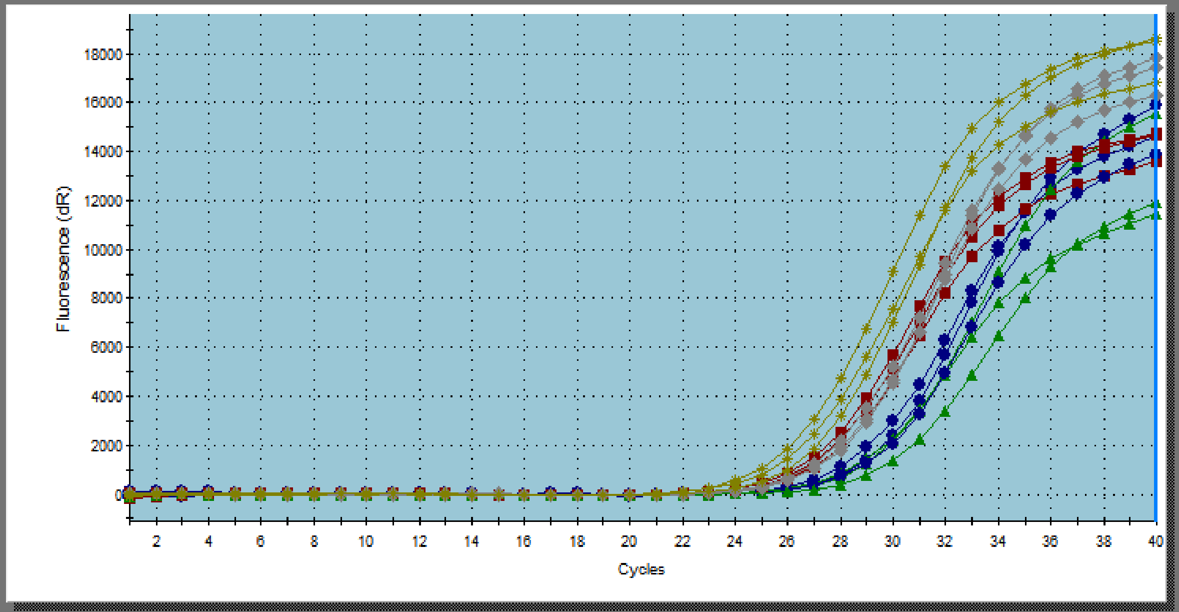

BCL2A

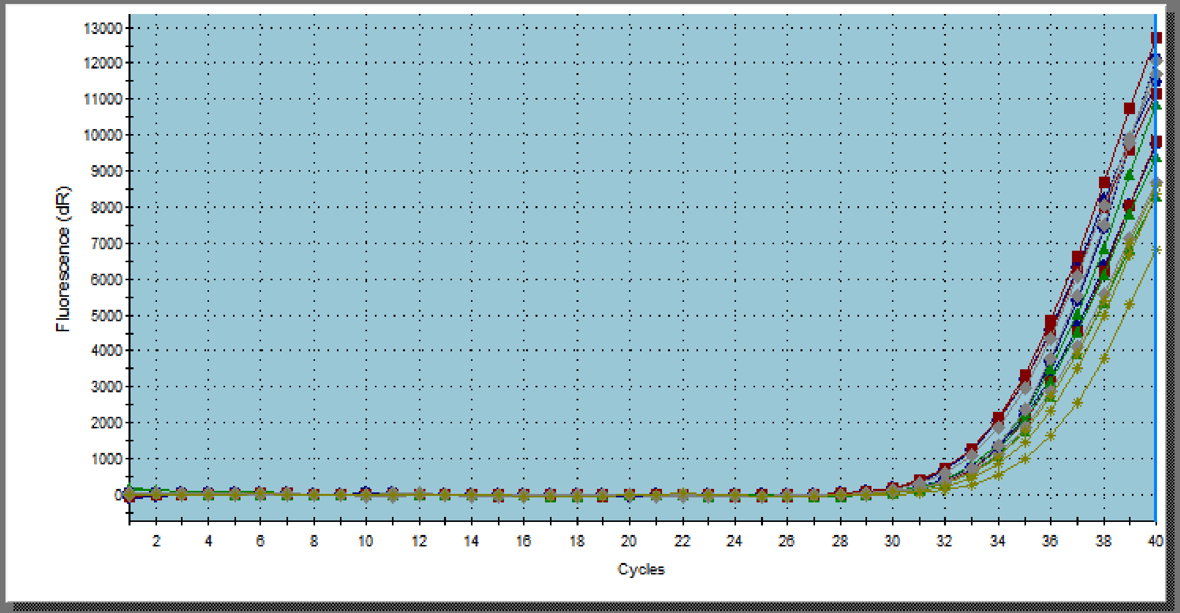

HMBS

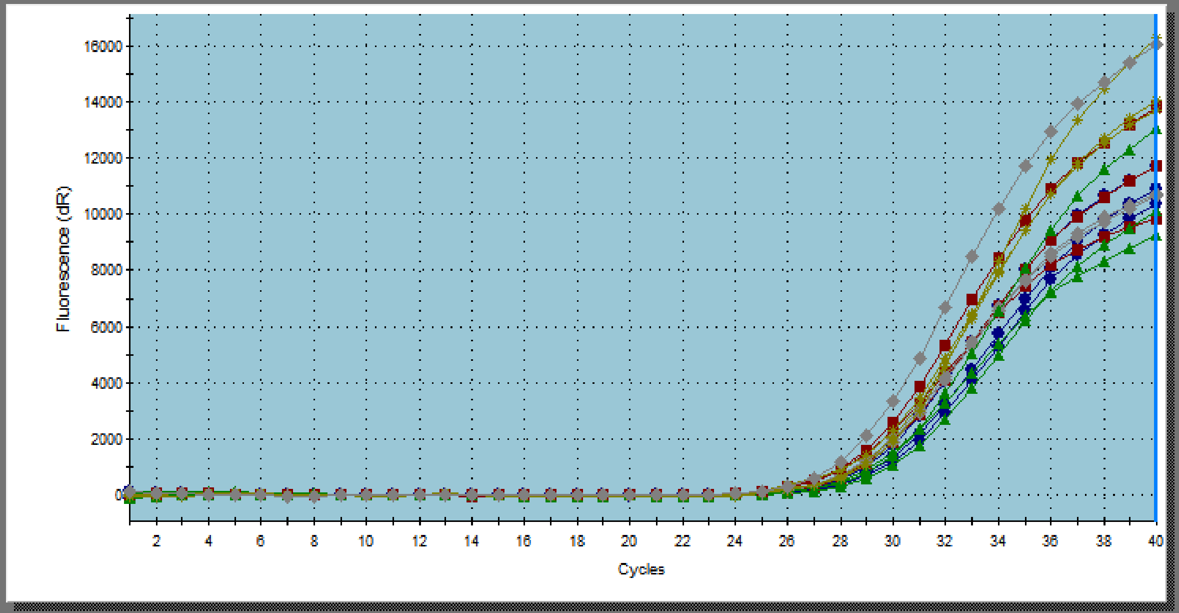

BAX

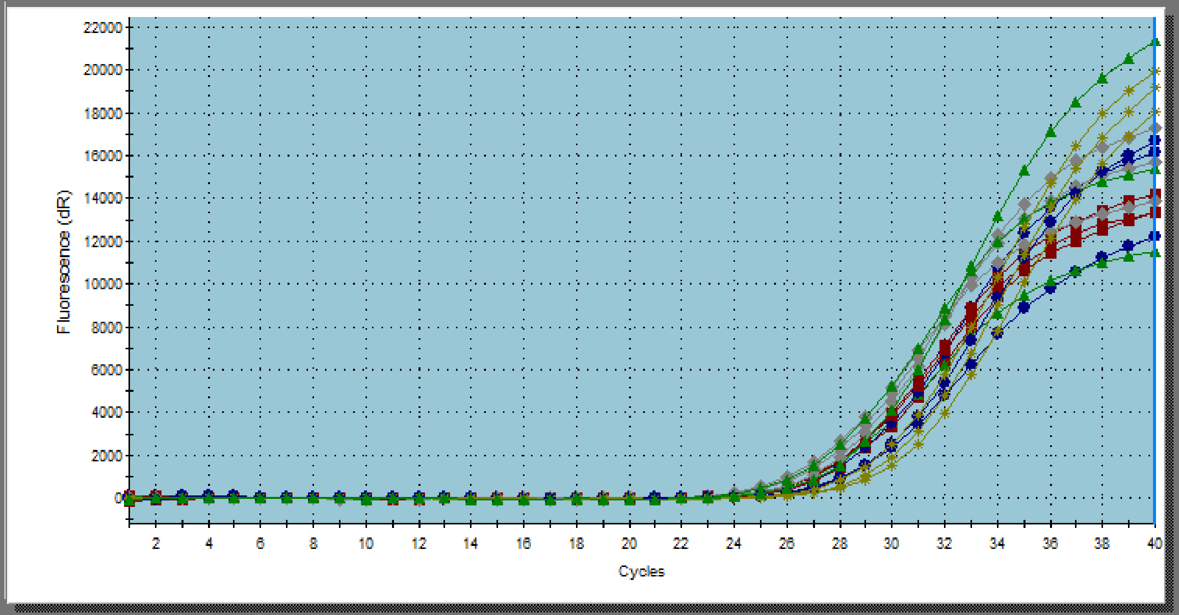
22000
20000
18000
16000
14000
12000
10000
8000
6000
4000
2000
0
Fluorescence (dR)
2
4
6
8
10
12
14
16
18
20
22
24
26
28
30
32
34
36
38
40
Cycles

SF3A1

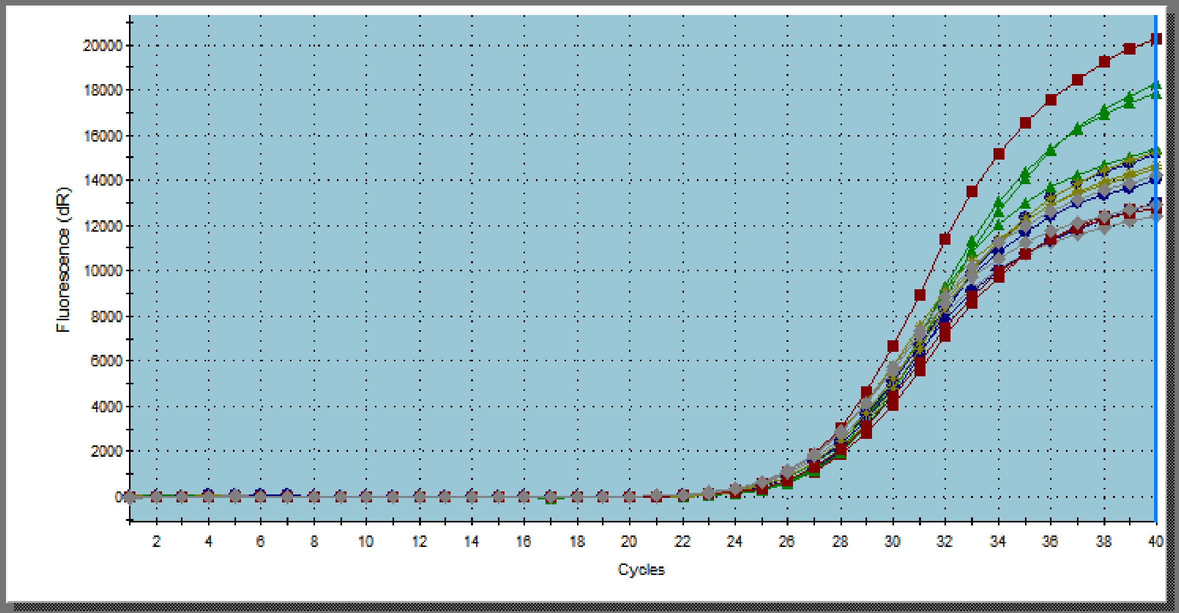
20000
18000
16000
14000
12000
10000
8000
6000
4000
2000
0
Fluorescence (dR)
2
4
6
8
10
12
14
16
18
20
22
24
26
28
30
32
34
36
38
40
Cycles

Experiment	Well	Well Name	Assay	Replicate	Ct (dR)	Rel. Quant. to Cal. (dR)
1. Experimento Combinados Cecilia_1.2	F3	ANY	BAX	3	25.89	Calibrator
2. Experimento Combinados_2 Cecilia	A3	ANY	BAX	3	24.49	Calibrator
4. Experimento Combinados BAX 31-05-	C1	ANY	BAX	3	25.21	Calibrator
1. Experimento Combinados Cecilia_1.2	A5	ANY	HMBS	5	27.54	Normalizer
1. Experimento Combinados Cecilia_1.2	A11	ANY	HMBS	5	27.13	Normalizer
1. Experimento Combinados Cecilia_1.2	F5	ANY	HMBS	5	26.74	Normalizer
2. Experimento Combinados_2 Cecilia	F6	ANY	SF3A1	6	22.98	Normalizer
3. Experimento Combinados_3 Cecilia	B1	ANY	SF3A1	6	23.29	Normalizer
3. Experimento Combinados_3 Cecilia	C1	ANY	SF3A1	6	23.54	Normalizer
1. Experimento Combinados Cecilia_1.2	C9	ANY+CHX	BAX	15	25.24	4.50
2. Experimento Combinados_2 Cecilia	C9	ANY+CHX	BAX	15	24.09	3.89
2. Experimento Combinados_2 Cecilia	H3	ANY+CHX	BAX	15	24.03	4.05
1. Experimento Combinados Cecilia_1.2	C11	ANY+CHX	HMBS	17	26.84	Normalizer
1. Experimento Combinados Cecilia_1.2	H5	ANY+CHX	HMBS	17	27.73	Normalizer
2. Experimento Combinados_2 Cecilia	H5	ANY+CHX	HMBS	17	26.75	Normalizer
2. Experimento Combinados_2 Cecilia	C6	ANY+CHX	SF3A1	18	26.49	Normalizer
3. Experimento Combinados_3 Cecilia	A3	ANY+CHX	SF3A1	18	26.15	Normalizer
4. Experimento Combinados BAX 31-05-	E4	ANY+CHX	SF3A1	18	24.99	Normalizer
1. Experimento Combinados Cecilia_1.2	E9	ANY+CHX+DMAP	BAX	27	24.82	7.34
2. Experimento Combinados_2 Cecilia	E3	ANY+CHX+DMAP	BAX	27	23.57	10.1
4. Experimento Combinados BAX 31-05-	G2	ANY+CHX+DMAP	BAX	27	24.34	9.91
4. Experimento Combinados BAX 31-05-	C6	ANY+CHX+DMAP	HMBS	29	29.64	Normalizer
4. Experimento Combinados BAX 31-05-	G5	ANY+CHX+DMAP	HMBS	29	29.71	Normalizer
4. Experimento Combinados BAX 31-05-	G6	ANY+CHX+DMAP	HMBS	29	29.91	Normalizer
4. Experimento Combinados BAX 31-05-	C4	ANY+CHX+DMAP	SF3A1	30	24.82	Normalizer
4. Experimento Combinados BAX 31-05-	G3	ANY+CHX+DMAP	SF3A1	30	25.52	Normalizer
4. Experimento Combinados BAX 31-05-	G4	ANY+CHX+DMAP	SF3A1	30	25.31	Normalizer
4. Experimento Combinados BAX 31-05-	B2	ANY+DMAP	BAX	9	25.79	6.66
4. Experimento Combinados BAX 31-05-	F1	ANY+DMAP	BAX	9	26.27	4.78
4. Experimento Combinados BAX 31-05-	F2	ANY+DMAP	BAX	9	25.22	9.88
1. Experimento Combinados Cecilia_1.2	B11	ANY+DMAP	HMBS	11	25.77	Normalizer
4. Experimento Combinados BAX 31-05-	B6	ANY+DMAP	HMBS	11	30.73	Normalizer

4. Experimento Combinados BAX 31-05-	F5	ANY+DMAP	HMBS	11	31.10	Normalizer
4. Experimento Combinados BAX 31-05-	B4	ANY+DMAP	SF3A1	12	25.48	Normalizer
4. Experimento Combinados BAX 31-05-	F3	ANY+DMAP	SF3A1	12	25.67	Normalizer
4. Experimento Combinados BAX 31-05-	F4	ANY+DMAP	SF3A1	12	26.28	Normalizer
2. Experimento Combinados_2 Cecilia	D3	CHX+DMAP	BAX	21	23.25	1.85
2. Experimento Combinados_2 Cecilia	D9	CHX+DMAP	BAX	21	23.22	1.89
2. Experimento Combinados_2 Cecilia	F9	CHX+DMAP	BAX	21	23.96	1.14
2. Experimento Combinados_2 Cecilia	D5	CHX+DMAP	HMBS	23	25.97	Normalizer
2. Experimento Combinados_2 Cecilia	D11	CHX+DMAP	HMBS	23	25.45	Normalizer
2. Experimento Combinados_2 Cecilia	F11	CHX+DMAP	HMBS	23	25.83	Normalizer
2. Experimento Combinados_2 Cecilia	D6	CHX+DMAP	SF3A1	24	23.41	Normalizer
2. Experimento Combinados_2 Cecilia	D12	CHX+DMAP	SF3A1	24	23.91	Normalizer
2. Experimento Combinados_2 Cecilia	F12	CHX+DMAP	SF3A1	24	23.78	Normalizer

Experiment	Well	Well Name	Assay	Replicate	Ct (dR)	Rel. Quant. to Cal. (dR)
1. Experimento Combinados Cecilia_1.2	A10	ANY	BCL2A	4	30.85	Calibrator
1. Experimento Combinados Cecilia_1.2	A5	ANY	HMBS	5	27.41	Normalizer
1. Experimento Combinados Cecilia_1.2	A11	ANY	HMBS	5	26.98	Normalizer
1. Experimento Combinados Cecilia_1.2	F5	ANY	HMBS	5	26.58	Normalizer
2. Experimento Combinados_2 Cecilia	A10	ANY	BCL2A	4	30.17	Calibrator
2. Experimento Combinados_2 Cecilia	F4	ANY	BCL2A	4	31.06	Calibrator
2. Experimento Combinados_2 Cecilia	A12	ANY	SF3A1	6	23.57	Normalizer
3. Experimento Combinados_3 Cecilia	B1	ANY	SF3A1	6	23.35	Normalizer
3. Experimento Combinados_3 Cecilia	C1	ANY	SF3A1	6	23.57	Normalizer
1. Experimento Combinados Cecilia_1.2	C4	ANY+CHX	BCL2A	16	31.35	0.656
1. Experimento Combinados Cecilia_1.2	C10	ANY+CHX	BCL2A	16	30.88	0.958
1. Experimento Combinados Cecilia_1.2	C11	ANY+CHX	HMBS	17	26.68	Normalizer
1. Experimento Combinados Cecilia_1.2	H5	ANY+CHX	HMBS	17	27.60	Normalizer
2. Experimento Combinados_2 Cecilia	C4	ANY+CHX	BCL2A	16	31.04	0.622
2. Experimento Combinados_2 Cecilia	H5	ANY+CHX	HMBS	17	27.11	Normalizer
2. Experimento Combinados_2 Cecilia	C12	ANY+CHX	SF3A1	18	22.88	Normalizer
2. Experimento Combinados_2 Cecilia	H6	ANY+CHX	SF3A1	18	23.53	Normalizer
3. Experimento Combinados_3 Cecilia	B3	ANY+CHX	SF3A1	18	23.61	Normalizer
1. Experimento Combinados Cecilia_1.2	E4	ANY+CHX+DMAP	BCL2A	28	31.20	0.457
1. Experimento Combinados Cecilia_1.2	E10	ANY+CHX+DMAP	BCL2A	28	32.60	0.147
1. Experimento Combinados Cecilia_1.2	E5	ANY+CHX+DMAP	HMBS	29	26.48	Normalizer
1. Experimento Combinados Cecilia_1.2	E11	ANY+CHX+DMAP	HMBS	29	26.06	Normalizer
2. Experimento Combinados_2 Cecilia	E4	ANY+CHX+DMAP	BCL2A	28	31.81	0.183
2. Experimento Combinados_2 Cecilia	E5	ANY+CHX+DMAP	HMBS	29	25.71	Normalizer
2. Experimento Combinados_2 Cecilia	E6	ANY+CHX+DMAP	SF3A1	30	22.87	Normalizer
1. Experimento Combinados Cecilia_1.2	B10	ANY+DMAP	BCL2A	10	31.08	0.590
1. Experimento Combinados Cecilia_1.2	B11	ANY+DMAP	HMBS	11	25.59	Normalizer
1. Experimento Combinados Cecilia_1.2	G5	ANY+DMAP	HMBS	11	26.55	Normalizer
2. Experimento Combinados_2 Cecilia	B4	ANY+DMAP	BCL2A	10	29.99	1.03
2. Experimento Combinados_2 Cecilia	G4	ANY+DMAP	BCL2A	10	29.95	1.06
2. Experimento Combinados_2 Cecilia	G5	ANY+DMAP	HMBS	11	25.78	Normalizer

3. Experimento Combinados_3 Cecilia	A2	ANY+DMAP	SF3A1	12	23.84	Normalizer
3. Experimento Combinados_3 Cecilia	B2	ANY+DMAP	SF3A1	12	23.26	Normalizer
3. Experimento Combinados_3 Cecilia	C2	ANY+DMAP	SF3A1	12	23.49	Normalizer
3. Experimento Combinados_3 Cecilia	A5	ANY+DMAP+CHX	SF3A1	30	23.04	Normalizer
3. Experimento Combinados_3 Cecilia	C5	ANY+DMAP+CHX	SF3A1	30	22.82	Normalizer
1. Experimento Combinados Cecilia_1.2	D4	CHX+DMAP	BCL2A	22	31.24	0.531
2. Experimento Combinados_2 Cecilia	D4	CHX+DMAP	BCL2A	22	30.25	0.926
2. Experimento Combinados_2 Cecilia	F10	CHX+DMAP	BCL2A	22	31.01	0.502
2. Experimento Combinados_2 Cecilia	D5	CHX+DMAP	HMBS	23	26.36	Normalizer
2. Experimento Combinados_2 Cecilia	D11	CHX+DMAP	HMBS	23	25.65	Normalizer
2. Experimento Combinados_2 Cecilia	F11	CHX+DMAP	HMBS	23	26.05	Normalizer
2. Experimento Combinados_2 Cecilia	D6	CHX+DMAP	SF3A1	24	23.29	Normalizer
2. Experimento Combinados_2 Cecilia	D12	CHX+DMAP	SF3A1	24	23.82	Normalizer
2. Experimento Combinados_2 Cecilia	F12	CHX+DMAP	SF3A1	24	23.69	Normalizer

Experiment	Well	Well Name	Assay	Replicate	Ct (dR)	Rel. Quant. to Cal. (dR)
1. Experimento Combinados Cecilia_1.2	A1	ANY	CDX2	1	26.77	Calibrator
1. Experimento Combinados Cecilia_1.2	A7	ANY	CDX2	1	26.81	Calibrator
1. Experimento Combinados Cecilia_1.2	F1	ANY	CDX2	1	26.99	Calibrator
1. Experimento Combinados Cecilia_1.2	A5	ANY	HMBS	5	27.41	Normalizer
1. Experimento Combinados Cecilia_1.2	A11	ANY	HMBS	5	26.98	Normalizer
1. Experimento Combinados Cecilia_1.2	F5	ANY	HMBS	5	26.58	Normalizer
2. Experimento Combinados_2 Cecilia	A12	ANY	SF3A1	6	23.57	Normalizer
3. Experimento Combinados_3 Cecilia	B1	ANY	SF3A1	6	23.35	Normalizer
3. Experimento Combinados_3 Cecilia	C1	ANY	SF3A1	6	23.57	Normalizer
1. Experimento Combinados Cecilia_1.2	C7	ANY+CHX	CDX2	13	25.79	2.17
1. Experimento Combinados Cecilia_1.2	H1	ANY+CHX	CDX2	13	27.29	0.727
1. Experimento Combinados Cecilia_1.2	C11	ANY+CHX	HMBS	17	26.68	Normalizer
1. Experimento Combinados Cecilia_1.2	H5	ANY+CHX	HMBS	17	27.60	Normalizer
2. Experimento Combinados_2 Cecilia	H1	ANY+CHX	CDX2	13	25.59	0.00
2. Experimento Combinados_2 Cecilia	H5	ANY+CHX	HMBS	17	27.11	Normalizer
2. Experimento Combinados_2 Cecilia	C12	ANY+CHX	SF3A1	18	22.88	Normalizer
2. Experimento Combinados_2 Cecilia	H6	ANY+CHX	SF3A1	18	23.53	Normalizer
3. Experimento Combinados_3 Cecilia	B3	ANY+CHX	SF3A1	18	23.61	Normalizer
1. Experimento Combinados Cecilia_1.2	E1	ANY+CHX+DMAP	CDX2	25	25.94	1.20
1. Experimento Combinados Cecilia_1.2	E7	ANY+CHX+DMAP	CDX2	25	25.34	1.86
1. Experimento Combinados Cecilia_1.2	G7	ANY+CHX+DMAP	CDX2	25	25.35	2.47
1. Experimento Combinados Cecilia_1.2	E5	ANY+CHX+DMAP	HMBS	29	26.48	Normalizer
1. Experimento Combinados Cecilia_1.2	E11	ANY+CHX+DMAP	HMBS	29	26.06	Normalizer
2. Experimento Combinados_2 Cecilia	E5	ANY+CHX+DMAP	HMBS	29	25.71	Normalizer
2. Experimento Combinados_2 Cecilia	E6	ANY+CHX+DMAP	SF3A1	30	22.87	Normalizer
1. Experimento Combinados Cecilia_1.2	B1	ANY+DMAP	CDX2	7	26.91	0.694
1. Experimento Combinados Cecilia_1.2	B7	ANY+DMAP	CDX2	7	26.21	1.15
1. Experimento Combinados Cecilia_1.2	G1	ANY+DMAP	CDX2	7	26.04	1.31
1. Experimento Combinados Cecilia_1.2	B11	ANY+DMAP	HMBS	11	25.59	Normalizer
1. Experimento Combinados Cecilia_1.2	G5	ANY+DMAP	HMBS	11	26.55	Normalizer
2. Experimento Combinados_2 Cecilia	G5	ANY+DMAP	HMBS	11	25.78	Normalizer

3. Experimento Combinados_3 Cecilia	A2	ANY+DMAP	SF3A1	12	23.84	Normalizer
3. Experimento Combinados_3 Cecilia	B2	ANY+DMAP	SF3A1	12	23.26	Normalizer
3. Experimento Combinados_3 Cecilia	C2	ANY+DMAP	SF3A1	12	23.49	Normalizer
3. Experimento Combinados_3 Cecilia	A5	ANY+DMAP+CHX	SF3A1	30	23.04	Normalizer
3. Experimento Combinados_3 Cecilia	C5	ANY+DMAP+CHX	SF3A1	30	22.82	Normalizer
1. Experimento Combinados Cecilia_1.2	D1	CHX+DMAP	CDX2	19	25.62	1.82
1. Experimento Combinados Cecilia_1.2	D7	CHX+DMAP	CDX2	19	26.24	1.15
1. Experimento Combinados Cecilia_1.2	F7	CHX+DMAP	CDX2	19	26.29	1.11
2. Experimento Combinados_2 Cecilia	D5	CHX+DMAP	HMBS	23	26.36	Normalizer
2. Experimento Combinados_2 Cecilia	D11	CHX+DMAP	HMBS	23	25.65	Normalizer
2. Experimento Combinados_2 Cecilia	F11	CHX+DMAP	HMBS	23	26.05	Normalizer
2. Experimento Combinados_2 Cecilia	D6	CHX+DMAP	SF3A1	24	23.29	Normalizer
2. Experimento Combinados_2 Cecilia	D12	CHX+DMAP	SF3A1	24	23.82	Normalizer
2. Experimento Combinados_2 Cecilia	F12	CHX+DMAP	SF3A1	24	23.69	Normalizer

Experiment	Well	Well Name	Assay	Replicate	Ct (dR)	Rel. Quant. to Cal. (dR)
1. Experimento Combinados Cecilia_1.2	A8	ANY	OCT.4	2	24.96	Calibrator
1. Experimento Combinados Cecilia_1.2	F2	ANY	OCT.4	2	25.58	Calibrator
1. Experimento Combinados Cecilia_1.2	A5	ANY	HMBS	5	27.41	Normalizer
1. Experimento Combinados Cecilia_1.2	A11	ANY	HMBS	5	26.98	Normalizer
1. Experimento Combinados Cecilia_1.2	F5	ANY	HMBS	5	26.58	Normalizer
2. Experimento Combinados_2 Cecilia	A8	ANY	OCT.4	2	25.98	Calibrator
2. Experimento Combinados_2 Cecilia	A12	ANY	SF3A1	6	23.57	Normalizer
3. Experimento Combinados_3 Cecilia	B1	ANY	SF3A1	6	23.35	Normalizer
3. Experimento Combinados_3 Cecilia	C1	ANY	SF3A1	6	23.57	Normalizer
1. Experimento Combinados Cecilia_1.2	C8	ANY+CHX	OCT.4	14	25.12	1.08
1. Experimento Combinados Cecilia_1.2	H2	ANY+CHX	OCT.4	14	25.54	0.830
1. Experimento Combinados Cecilia_1.2	C11	ANY+CHX	HMBS	17	26.68	Normalizer
1. Experimento Combinados Cecilia_1.2	H5	ANY+CHX	HMBS	17	27.60	Normalizer
2. Experimento Combinados_2 Cecilia	H2	ANY+CHX	OCT.4	14	27.18	0.436
2. Experimento Combinados_2 Cecilia	H5	ANY+CHX	HMBS	17	27.11	Normalizer
2. Experimento Combinados_2 Cecilia	C12	ANY+CHX	SF3A1	18	22.88	Normalizer
2. Experimento Combinados_2 Cecilia	H6	ANY+CHX	SF3A1	18	23.53	Normalizer
3. Experimento Combinados_3 Cecilia	B3	ANY+CHX	SF3A1	18	23.61	Normalizer
1. Experimento Combinados Cecilia_1.2	E5	ANY+CHX+DMAP	HMBS	29	26.48	Normalizer
1. Experimento Combinados Cecilia_1.2	E11	ANY+CHX+DMAP	HMBS	29	26.06	Normalizer
2. Experimento Combinados_2 Cecilia	E2	ANY+CHX+DMAP	OCT.4	26	23.67	2.14
2. Experimento Combinados_2 Cecilia	E8	ANY+CHX+DMAP	OCT.4	26	22.69	3.93
2. Experimento Combinados_2 Cecilia	G8	ANY+CHX+DMAP	OCT.4	26	22.76	3.76
2. Experimento Combinados_2 Cecilia	E5	ANY+CHX+DMAP	HMBS	29	25.71	Normalizer
2. Experimento Combinados_2 Cecilia	E6	ANY+CHX+DMAP	SF3A1	30	22.87	Normalizer
1. Experimento Combinados Cecilia_1.2	B8	ANY+DMAP	OCT.4	8	23.71	1.88
1. Experimento Combinados Cecilia_1.2	B11	ANY+DMAP	HMBS	11	25.59	Normalizer
1. Experimento Combinados Cecilia_1.2	G5	ANY+DMAP	HMBS	11	26.55	Normalizer
2. Experimento Combinados_2 Cecilia	B8	ANY+DMAP	OCT.4	8	23.63	2.82
2. Experimento Combinados_2 Cecilia	G2	ANY+DMAP	OCT.4	8	24.11	2.10
2. Experimento Combinados_2 Cecilia	G5	ANY+DMAP	HMBS	11	25.78	Normalizer

3. Experimento Combinados_3 Cecilia	A2	ANY+DMAP	SF3A1	12	23.84	Normalizer
3. Experimento Combinados_3 Cecilia	B2	ANY+DMAP	SF3A1	12	23.26	Normalizer
3. Experimento Combinados_3 Cecilia	C2	ANY+DMAP	SF3A1	12	23.49	Normalizer
3. Experimento Combinados_3 Cecilia	A5	ANY+DMAP+CHX	SF3A1	30	23.04	Normalizer
3. Experimento Combinados_3 Cecilia	C5	ANY+DMAP+CHX	SF3A1	30	22.82	Normalizer
2. Experimento Combinados_2 Cecilia	D2	CHX+DMAP	OCT.4	20	24.48	1.86
2. Experimento Combinados_2 Cecilia	D8	CHX+DMAP	OCT.4	20	24.43	1.91
2. Experimento Combinados_2 Cecilia	F8	CHX+DMAP	OCT.4	20	24.27	2.12
2. Experimento Combinados_2 Cecilia	D5	CHX+DMAP	HMBS	23	26.36	Normalizer
2. Experimento Combinados_2 Cecilia	D11	CHX+DMAP	HMBS	23	25.65	Normalizer
2. Experimento Combinados_2 Cecilia	F11	CHX+DMAP	HMBS	23	26.05	Normalizer
2. Experimento Combinados_2 Cecilia	D6	CHX+DMAP	SF3A1	24	23.29	Normalizer
2. Experimento Combinados_2 Cecilia	D12	CHX+DMAP	SF3A1	24	23.82	Normalizer
2. Experimento Combinados_2 Cecilia	F12	CHX+DMAP	SF3A1	24	23.69	Normalizer
